# Effect of experimental knee pain location on gait kinematics

**DOI:** 10.1007/s00421-024-05648-3

**Published:** 2024-11-14

**Authors:** Alessio Gallina, Paola Adamo, Giorgia Marino, Corrado Cescon, Francesco Bolzoni, Marco Barbero, Federico Temporiti, Roberto Gatti

**Affiliations:** 1 School of Sport, Exercise and Rehabilitation Sciences, College of Life and Environmental Sciences, University of Birmingham, Birmingham, UK; 2 Physiotherapy Unit, IRCCS Humanitas Research Hospital, Rozzano, Italy; 3 Department of Biomedical Sciences, Humanitas University, Pieve Emanuele, Italy; 4 Rehabilitation Research Laboratory 2rLab, Department of Business Economics, Health and Social Care (DEASS), University of Applied Sciences and Arts of Southern Switzerland (SUPSI), Manno, Switzerland

**Keywords:** Pain, Gait, Knee, Electrical stimulation, Kinematics, Arthrokinematics

## Abstract

**Purpose:**

In this study, we investigated whether experimental knee pain alters lower limb kinematics and knee arthrokinematics during gait, and if this motor adaptation depends on the spatial characteristics of the painful stimulus.

**Methods:**

Twenty-one participants walked on a treadmill for 60-s trials, either without stimulation or while experiencing painful electrical stimulation in the medial, lateral or anterior region of the knee. Perceived pain location was analyzed using pain drawing. Gait spatiotemporal parameters, lower limb kinematics, and dispersion of the knee helical axes on the sagittal plane were quantified for each trial and compared between conditions using ANOVAs with repeated measures or Friedman tests.

**Results:**

Pain perception was localized in the area the stimulation was applied to. Compared to walking without pain, participants demonstrated reduced knee extension (1.5 ± 1.5 degrees, *p* = 0.002) and reduced hip extension (0.8 ± 1.1 degrees, *p* = 0.037) when pain was induced in the anterior region, but not medially or laterally. Anterior knee pain increased the mean distance of the helical axes during late stance (0.7 [0.3, 1.4], *p* = 0.010), while medial pain increased both mean distance (0.3 [0.1, 0.5], *p* = 0.037) and mean angle (1.2 ± 1.4, *p* = 0.010) during early swing.

**Conclusion:**

Acute, experimental knee pain alters gait kinematics and increases the dispersion of the helical axis. These adaptations depend on the spatial characteristics of the painful stimulus. These adaptations may reflect an attempt of the central nervous system to protect the painful tissue while searching for a less painful movement strategy.

## Introduction

Current theories predict that, in response to a painful stimulus, the central nervous system changes movement strategies to limit stress on the painful/injured tissues (Hodges and Tucker 2011). These adaptations may range from movement avoidance to subtle changes in the way a task is performed (Hodges and Smeets 2015). When considering gait, a variety of adaptations have been described in response to acute anterior knee pain. Injections or infusion of hypertonic saline solution in the infrapatellar fat pad resulted in decreased plantar flexion peak (Seeley et al. 2013), increased knee flexion at initial contact (Son et al. 2017), and increased knee flexion during stance (Henriksen et al. 2010). Some of these adaptations are similar to those observed after joint infusion (Torry et al. 2000) and to the gait kinematics of individuals with tibiofemoral osteoarthritis (Henriksen et al. 2010). While adaptations of gait biomechanics in response to experimental anterior knee pain are well described, it is unclear whether these adaptations depend on the spatial characteristics of the pain. In addition, it is currently unclear whether experimental knee pain results in subtle alterations of arthrokinematics in addition to changes of joint kinematics.

Individuals with knee musculoskeletal disorders report pain in different regions around the knee joint. Patellofemoral pain commonly results in pain around/behind the patella (Collins et al. 2018), although with large inter-individual variability (Matthews et al. 2018), whereas iliotibial band syndrome results in pain localized on the lateral side of the knee (Khaund and Flynn 2005). Different spatial characteristics of pain have also been described in individuals with tibiofemoral osteoarthritis, with predominant patterns of diffused patellar pain, or localized pain in the medial or lateral side of the knee (Thompson et al. 2009). While individuals with knee musculoskeletal disorders present with variable pain characteristics, it is currently unknown whether different spatial characteristics of perceived pain result in different motor adaptations.

Most research investigating motor adaptation to experimental knee pain induced pain by injecting hypertonic saline solution into the infrapatellar fat pad (Bennell et al. 2004; Hodges et al. 2009; Tucker and Hodges 2009; Henriksen et al. 2011a; Seeley et al. 2013; Shiozawa et al. 2015), which recreates a sensation of diffuse pain around/behind the patella (Bennell et al. 2004). To our knowledge, only a few studies directly compared whether motor adaptation differs when pain is induced in different locations around the knee. For instance, injection of hypertonic saline solution in the distal vastus medialis altered quadriceps muscle activation, whereas injection of the infrapatellar fat pad selectively changed knee extension force direction (Gallina et al. 2018). When pain was induced using hypertonic saline solution in the medial collateral ligament or in the iliotibial tract, knee extension strength decreased more after injection of the medial collateral ligament (Oda et al. 2018). While there is preliminary evidence that pain location may drive motor adaptation in isometric tasks, it is currently unknown whether the spatial characteristics of pain drive biomechanics adaptation during daily living activities, such as gait.

Besides changing joint kinematics, pain may also result in subtle changes in arthrokinematics. By inferring joint rotation center displacements during movements, helical axes dispersion represents an index of joint stability and movement control. Helical axes dispersion has been shown to differ between young and elderly participants (Temporiti et al. 2019), increase with shoulder muscle fatigue (Adamo et al. 2020) and differ across gait phases at the level of the knee joint (Temporiti et al. 2020). When comparing individuals with and without chronic musculoskeletal disorders, helical axes are more irregular in people with neck pain or who sustained a whiplash injury (Grip et al. 2008), and dispersion of the helical axes is smaller in individuals with neck pain, which suggests a decreased motor variability (Alsultan et al. 2019; Cattrysse et al. 2020). Whether the dispersion of helical axes changes during acute, experimental pain remains to be determined.

In this study, we investigated whether experimental knee pain alters lower limb kinematics and knee arthrokinematics during gait, and if this motor adaptation differs when pain is induced in the medial, anterior, or lateral region of the knee. We hypothesized that acute experimental pain would alter spatiotemporal gait parameters, lower limb kinematics (characterized as joint angles) and arthrokinematics (characterized as dispersion of the helical axes), and that these changes would depend on the spatial characteristics of the painful stimulus.

## Methods

### Participants

Twenty-one asymptomatic participants (5 females, 16 males; age: 25.0 ± 5.3 years; height: 176.3 ± 6.6 kg; weight: 71.1 ± 10.5 kg) participated in the study. To be eligible to take part in the study, participants had to be 18–40 years old. People who reported current lower limb pain, major pathologies that affect lower limb mobility or balance, or who had undergone lower limb surgery, were ineligible to participate. The study was approved by the Internal Ethical Committee of Humanitas Research Hospital (code: CLF22/02) and participants signed a written informed consent prior to data collection.

### Protocol

**Fig. 1:**
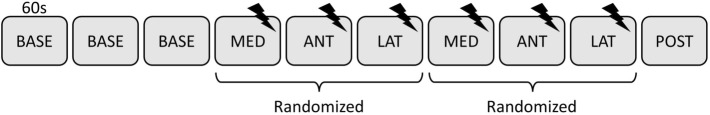
Protocol. Each block identifies a 60-s gait trial. *Base* baseline, no stimulation, *Med* medial stimulation, *Ant* anterior stimulation, *Lat* lateral stimulation, *Post* after the painful trials, no stimulation

After reporting which leg participants would use kick a ball to establish leg dominance (Melick et al. 2017), participants identified their self-selected walking speed on a treadmill in a familiarization trial. Each participant completed ten trials of at least 60 s of gait at the self-selected speed. The protocol is illustrated visually in Fig. 1. Participants performed three trials without painful stimulation to obtain stable baseline data. Then, they walked while experiencing painful stimulation in six trials, with pain locations randomized. Finally, they performed one trial without painful stimulation to assess whether gait adaptations outlasted the painful stimulation. At least 1 min of rest was provided between trials.

Pain was induced in the right knee by means of electrical stimulation (Tucker et al. 2012; Gallina et al. 2021; Cabral et al. 2023). This methodology was preferred to other models such as injections of hypertonic saline solution because the pain location, intensity and duration can be easily controlled and standardized across participants. We have previously demonstrated that painful electrical stimulation reduces maximal knee extension strength, and that the decrease in force production is comparable to that of previous studies using hypertonic saline solution injections (Cabral et al. 2023). Three pairs of surface electrodes (I-Tech, 30 mm) were placed over the skin of the medial, lateral and anterior knee joint. Anteriorly, the electrodes were placed on the medial and lateral edges of the patella, approximately 6.0 ± 0.8 cm apart. This location was chosen to reproduce widespread pain behind/around the patella, commonly reported by individuals with patellofemoral pain (Collins et al. 2018). Medially and laterally, the electrodes were placed bridging the medial tibial condyle or the lateral femoral condyle in the antero-posterior direction. For both locations, the interelectrode distance was 2.1 ± 0.2 cm. The smaller interelectrode distance was necessary to avoid stimulation of muscle fibers of the distal vastus medialis and lateralis muscles. In addition, previous research (Thompson et al. 2009) showing that individuals with tibiofemoral osteoarthritis report either localized pain on the medial or lateral joint line, or regional pain around the patella, further supports the larger interelectrode distance for the anterior compared to the medial and lateral locations. Electrical stimulation was applied using an electrical stimulator (DS7, Digitimer, UK), controlled using a NIDAQ board (National Instruments, DAQ 782602-01) and Matlab (version, R2022b). The waveform was a 100-µs-long biphasic square wave, delivered at 20 Hz. Before the baseline trials, we determined the intensity of the stimulation needed to induce the target level of pain by incrementally increasing the stimulation intensity while asking the participant to report the intensity of the pain experienced on a Numerical Rating Scale (0–10 points, 0: no pain, 10: maximum pain). We applied 1-s-long stimuli starting from 0 mA, increasing in steps of 3 mA until the participant reported a pain of at least 3/10. Then, the stimulation intensity was increased in steps of 0.5 mA to identify the stimulation intensity necessary to induce a pain sensation of 4 out of 10. The stimulation intensity was then confirmed before the start of each trial while the participant stood on the treadmill. In each trial when pain was induced, the stimulation began before the participant started to walk on the treadmill. Participants reported the amount of pain 5 s after they started to walk at the self-selected speed, and at the end of the trial. At the end of each trial, participants drew the location where they felt pain on a body chart depicting the cross-section of a knee (Gallina et al. 2021). As a qualitative assessment of the temporal characteristics of the pain, participants were asked to report whether their pain increased during a specific phase of the gait cycle.

Lower limb kinematics was collected using an optical motion capture system (SMART-DX, BTS, Italy) using a sampling rate at 100 Hz. Four clusters of 5 retro-reflective markers were fixed to the lateral surface of the right thigh and shank using inextensible bands (Temporiti et al. 2020) to minimize skin motion artifacts. Eleven retro-reflective markers were placed on anatomical landmarks of the pelvis and right leg following Helen Hayes protocol (Kadaba et al. 1990), and the anthropometric measures needed to calculate kinematics were collected.

### Data processing

Lower limb kinematics was calculated using SmartTracker (BTS Bioengineering, Italy), and further processed using Matlab (R2021a, Mathworks, USA). For each trial, 60 s of gait at steady self-selected speed were analyzed. Individual gait cycles were identified based on the location of the marker on the heel. Hip, knee and ankle joint angles were calculated as Euler angles (XYZ sequence), low-pass filtered at 5 Hz (Butterworth, 2nd order), then averaged across all gait cycles. We also calculated the number of strides per minute (cadence), the time between two consecutive heel strikes (gait cycle duration), the antero-posterior displacement of the heel marker between toe off and heel strike (stride length), and the time between heel strike and toe off expressed as percentage of the gait cycle duration (stance phase duration).

For the calculation of the helical axes, raw marker data were low-pass filtered at 5 Hz (Butterworth, 2nd order). Markers clusters were used to identify tights and shanks as rigid bodies, and their mutual positions were described every 10 degrees of motion along the sagittal plane as a composition of a rotation around and translation along a fixed axis using the methodology described previously (Temporiti et al. 2020; Adamo et al. 2022). Knee helical axes dispersion was described using the mean distance and mean angle parameters in order to quantify helical axes displacement and orientation during the gait cycle. Mean distance represents the distance among helical axes providing a measure of joint rotation center displacement, whereas mean angle consists of a measure of helical axes orientation and quantifies the plane of motion variability during the joint movements. Mean distance and mean angle are presented for the sagittal plane which was the plane of motion that demonstrated highest reliability (intraclass correlation coefficients between 0.70 and 0.90; (Adamo et al. 2022)). Each gait cycle was divided into the following phases: (1) early stance, from 95% of the previous gait cycle to 10% of the subsequent gait cycle; (2) late stance, from 10 to 40% of gait cycle; (3) early swing, from 40 to 70% of gait cycle; and (4) Late swing, from 70 to 95% of gait cycle (Temporiti et al. 2020). For both joint angles and helical axes, estimates obtained in multiple repetitions of the same condition were averaged, resulting in a single value for baseline, anterior, medial, lateral, and after pain conditions.

Pain drawings were digitized as.jpg images and imported into Matlab. The colored pixels were discriminated from the white background and the black outline of the knee using the K-means algorithm. Since each pain condition was repeated twice, any pixel colored in at least one of the two maps was considered part of the colored area. The size of the colored area was calculated as the percentage of colored pixels with respect to the number of pixels contained within the knee cross-section outline.

Stimulation intensity and pain ratings were averaged across repetitions with the same pain location. Qualitative reports of whether pain was more intense in a specific phase of the gait cycle were classified in the following categories: ‘constant’, ‘stance’, ‘swing’, ‘toe off’, ‘heel strike’; each one indicates higher pain during that specific gait phase compared to the rest of the gait cycle (except ‘constant’).

All statistical analyses were performed using SPSS (version 28.0, IBM, Chicago). Parametric (one-way repeated measure ANOVAs) or non-parametric statistics (Friedman tests) with the condition as intra-subject variable were used based on data distribution (Shapiro–Wilk test). When Mauchly’s test identified violation of the sphericity assumption, a Greenhouse–Geisser correction was applied. In post-hoc comparisons, *p* values were Bonferroni-corrected for the number of comparisons. Depending on the distribution, data are presented either as mean and standard deviation, or as median and interquartile range. Stimulation intensity, pain intensity at the start and end of the trial, and all kinematic outcomes were compared across pain locations using ANOVAs for repeated measures or the Friedman tests. Pairwise comparisons were used as post-hoc tests. For each condition, pain intensity at the start and end of the trial was compared using a paired Wilcoxon signed-rank test.

## Results

### Pain characteristics

The average self-selected speed was 3.5 ± 0.4 m/s and 3 participants reported left as their dominant leg. The stimulation intensity necessary to induce a pain of 4/10 did not differ across locations (Table 1, F(2,40) = 0.952, *p* = 0.394). There was a trend for participants to report higher pain intensity when the painful stimulation was applied in the anterior location at the start of the trial (*χ*^2^(2) = 5.920, *p* = 0.052), but the reported pain intensity did not differ by the end of the trial (*χ*^2^(2) = 0.886, *p* = 0.642). Reported pain intensity did not consistently decrease throughout the trial when pain was induced in the medial (change: −0.25 [−0.5, 0], *Z* = 36.550, *p* = 0.099), anterior (change: −0.5 [−0.5, 0], *Z* = 33.500, *p* = 0.068), or lateral location (change: 0 [−0.5, 0], *Z* = 16.500, *p* = 0.073). Across the three stimulation conditions, participants reported higher pain during stance in 29.4% of the trials, during swing in 27.8%, constant pain throughout the gait cycle in 26.2%, and higher pain during heel strike and toe off in 8.7% and 7.9% of the trials. Frequencies by pain location are shown in Table 1. For each pair of trials where the painful stimulation was applied to the same location, participants reported pain in the same gait phase in 77.8% of the cases.

**Table 1:** Stimulation intensity and pain characteristics

	Medial	Anterior	Lateral
Stimulation intensity (mA)	18.2 ± 4.7	16.7 ± 4.8	17.9 ± 4.4
Pain intensity, start (out of 10)	4 [3.5, 4]	4 [4, 4]	4 [3.5, 4]
Pain intensity, end (out of 10)	3.5 [3, 4]	3.5 [3.5, 4.5]	3.5 [3, 4]
Pain location, area (%)	5.3 ± 3.9^a^	8.0 ± 4.5^l,m^	3.5 ± 2.4^a^
Temporal descriptors (% of trials)	Constant: 19.0% Heel strike: 0% Stance: 35.7% Swing: 38.1% Toe off: 7.1%	Constant: 31.0% Heel strike: 14.3% Stance: 23.8% Swing: 23.8% Toe off: 7.1%	Constant: 28.6% Heel strike: 11.9% Stance: 28.6% Swing: 21.4% Toe off: 9.5%

^a^different from anterior

^l^different from lateral

^m^different from medial

**Fig. 2:**
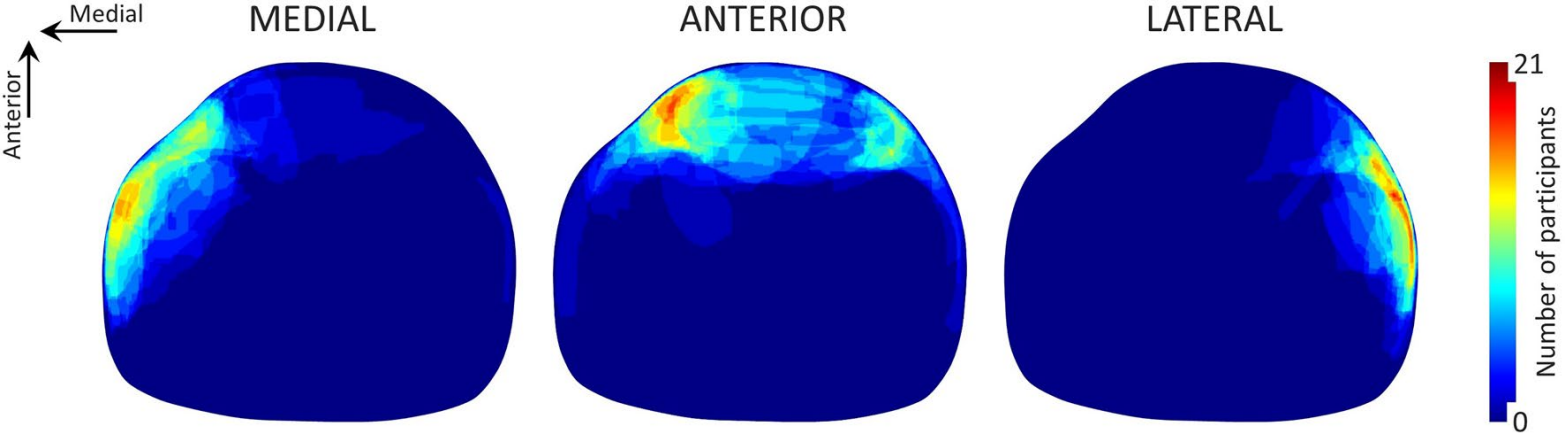
Pain drawing, cross-section of the knee (patella on top). Each heatmap illustrates the pain localization when painful stimulation was applied to the medial, anterior and lateral side of the knee. The color of each pixel identifies the number of participants who reported pain in that region of the knee

Pain drawings (Fig. 2) demonstrated that pain was perceived mainly in the area around the stimulation electrodes, confirming that the protocol used was able to elicit localized pain. Pain area (F(2,40) = 16.057, *p* < 0.001) and differed across pain locations. Patellar stimulation resulted in a pain area larger than that reported when stimulation was applied medially (*p* = 0.008) and twice as large as that reported during lateral stimulation (*p* < 0.001). The size of the area identified as painful was similar when the painful stimulus was applied medially or laterally (*p* = 0.085).

### Gait parameters and lower limb kinematics

Neither cadence (F(2.7,54.0) = 0.304, *p* = 0.802, Table 2) nor step length (F(2.8,55.7) = 2.06, *p* = 0.102) differed across conditions. Despite a significant main effect of painful stimulation on the duration of the stance phase (*χ*^2^(2) = 18.02, *p* = 0.001), post-hoc comparisons showed no significant differences between conditions after Bonferroni correction (*p* > 0.063).

**Table 2:** Gait parameters and lower limb kinematics. For kinematics, negative values indicate opposite directions; for instance, −10 degrees of hip adduction indicates 10 degrees of hip abduction

	Baseline	Medial	Anterior	Lateral	Post	*p*
Cadence (steps/min)	51.1 ± 4.4	50.9 ± 4.4	51.0 ± 4.5	51.2 ± 4.6	51.0 ± 4.5	0.802
Step length (cm)	63.1 ± 3.4	63.2 ± 3.8	62.7 ± 3.8	62.9 ± 4.3	63.6 ± 4.1	0.120
Stance duration (%)	61.2 ± 1.2	61.0 ± 1.5	60.9 ± 1.0	60.9 ± 1.0	61.5 ± 1.2	0.001; /
Hip flexion (deg)						
Max	37.7 ± 6.7	38.1 ± 6.8	38.1 ± 7.2	38.1 ± 6.6	38.5 ± 6.5	0.051; *P* > *B*
Min	−3.1 ± 7.0	−2.8 ± 7.0	−2.4 ± 7.6	−3.0 ± 7.1	−3.1 ± 7.4	0.009; *A* > *BL*
Hip adduction (deg)						
Max	0.9 ± 2.9	0.9 ± 3.1	0.8 ± 3.2	0.8 ± 3.2	1.3 ± 3.1	0.001; *P* > *AL*
Min	−10.2 ± 3.5	−10.1 ± 3.7	−10.0 ± 3.6	−10.3 ± 3.6	−10.2 ± 3.8	0.239
Hip internal rotation (deg)						
Max	14.1 ± 7.0	14.0 ± 7.5	13.8 ± 7.3	14.0 ± 7.6	14.2 ± 7.8	0.500
Min	3.5 ± 5.9	3.6 ± 6.5	3.4 ± 6.5	3.6 ± 6.5	3.2 ± 6.8	0.425
Knee flexion (deg)						
Max	65.2 ± 4.2	65.5 ± 5.3	65.0 ± 5.5	65.6 ± 5.4	66.6 ± 4.6	0.020; *P* > *BM*
Min	5.5 ± 4.6	6.0 ± 4.9	7.0 ± 4.8	6.1 ± 4.9	5.7 ± 4.6	< 0.001; *A* > *BMLP*
Ankle flexion (deg)						
Max	16.2 ± 4.2	16.6 ± 4.5	16.6 ± 4.4	16.7 ± 4.6	16.7 ± 4.6	0.365
Min	−14.9 ± 6.1	−14.5 ± 5.9	−13.9 ± 5.6	−14.6 ± 7.1	−14.8 ± 6.1	0.310

*A* anterior, *B* baseline, *L* lateral, *M* medial, *P* post

**Fig. 3:**
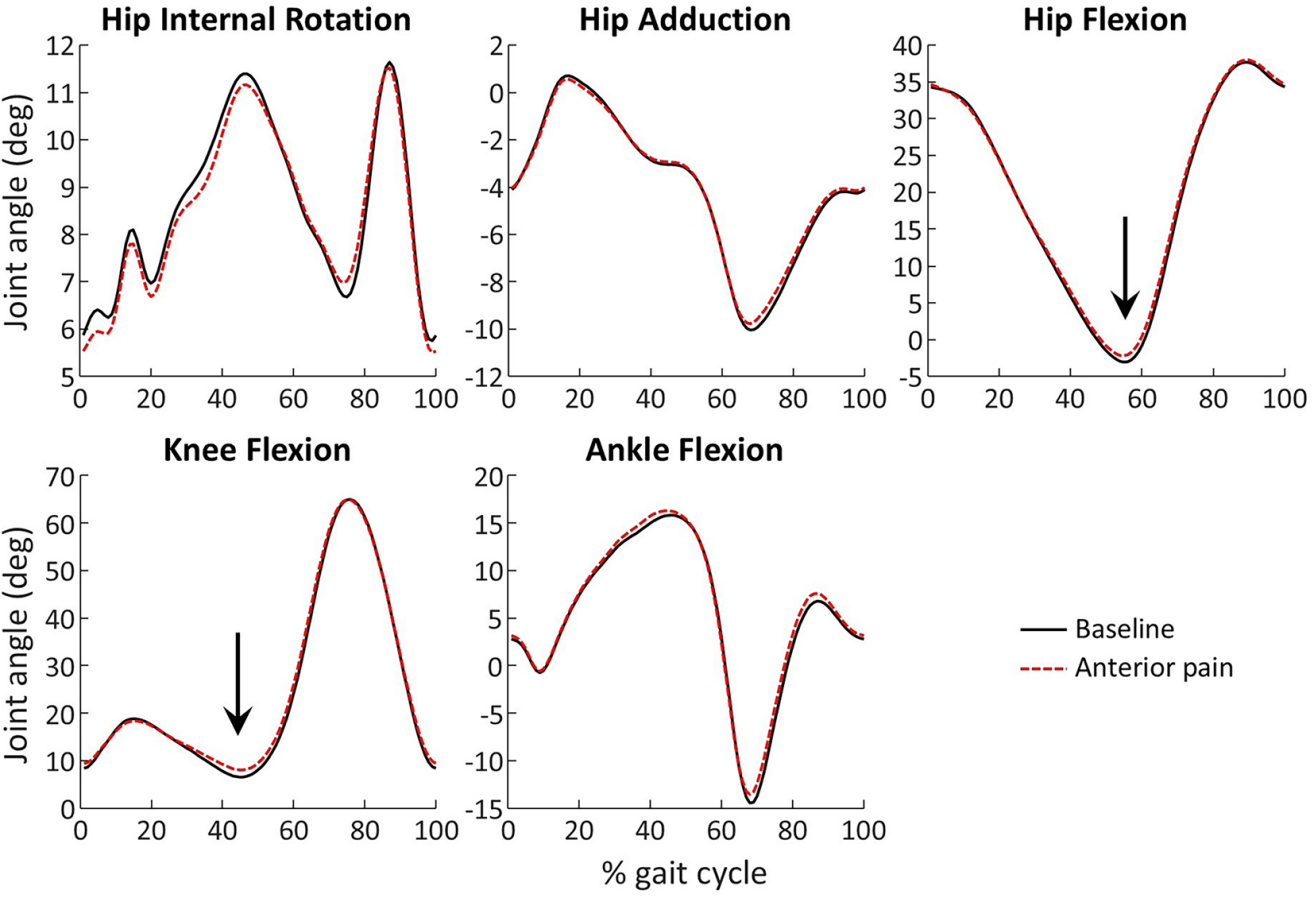
Kinematic waveforms at baseline and during anterior knee painful stimulation, average across 21 participants. Kinematics was normalized between heel strikes. The subplot title indicates what positive values represent. The arrows identify a significant decreased in hip and knee extension during the push-off phase

Ankle kinematic data from 2 participants was of low quality and had to be excluded, leaving 19 participants for the analysis of ankle kinematics. Average kinematics waveforms are shown in Fig. 3.

**Fig. 4:**
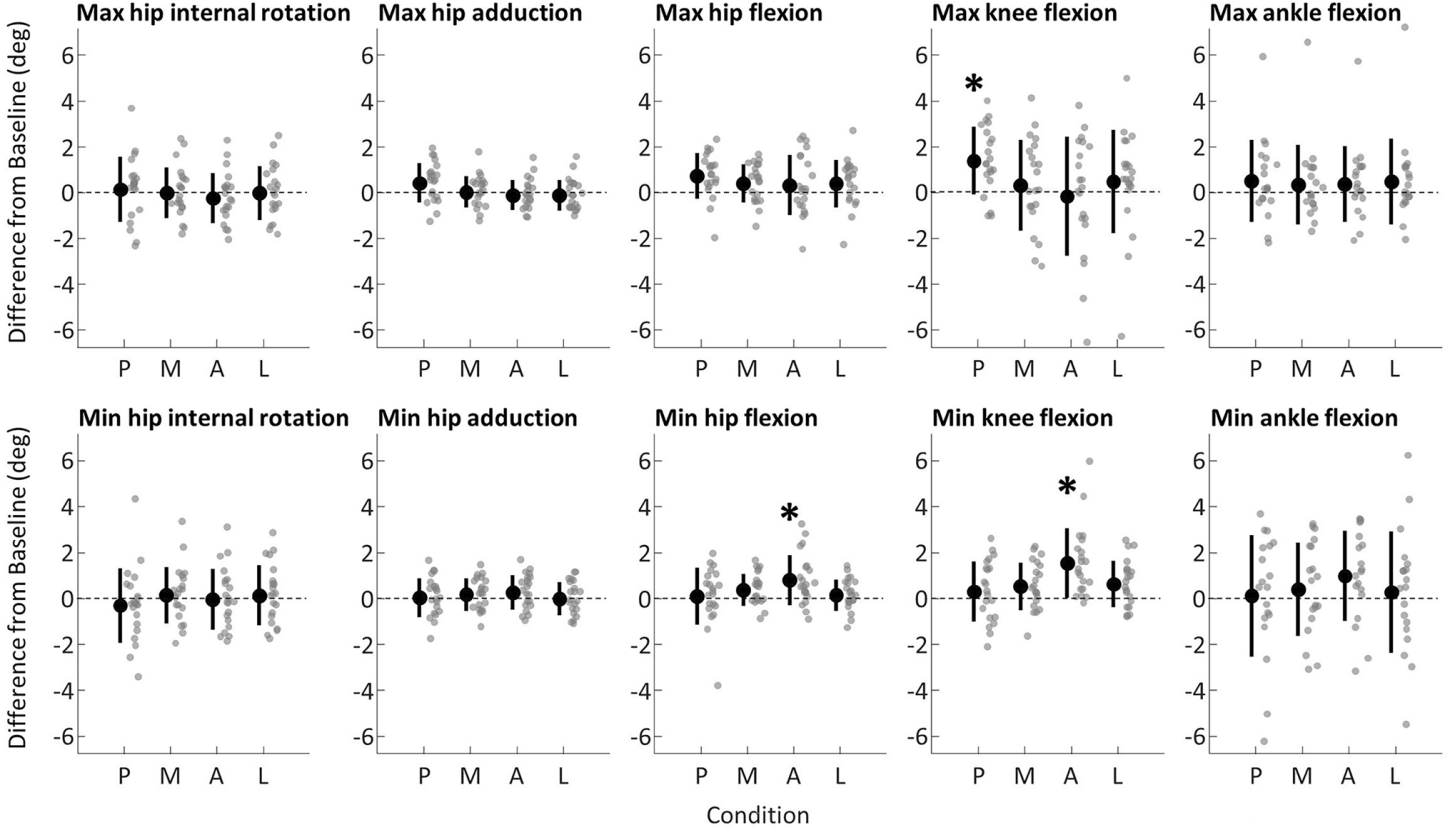
Comparison of lower limb kinematics. *P* post, *A* anterior, *B* baseline, *L* lateral; *M* medial. Data are presented as differences from baseline; ‘Max’ identifies the positive peak, whereas ‘Min’ identifies the negative peak. Gray dots identify individual participants, black circles and bars identify mean and standard deviation. Compared to baseline, participants walked with reduced knee and hip extension when pain was induced in the anterior region of the knee, and with increased knee flexion in the last trial without painful stimulation

Anterior knee pain mainly altered peak knee (F(2.9,59.3) = 9.778, *p* < 0.001, Fig. 4) and hip (F(2.7,54.3) = 4.393, *p* = 0.009) extension. Participants walked with less knee extension when pain was induced on the patella compared to baseline (1.5 ± 1.5 degrees, *p* = 0.002), post (1.2 ± 1.4 degrees, *p* = 0.005), medial pain (1.0 ± 1.3 degrees, *p* = 0.015) and lateral pain (0.6 ± 1.0 degrees, *p* = 0.016). Participants also walked with less hip extension when experiencing anterior knee pain compared to baseline (0.8 ± 1.1 degrees, *p* = 0.037) and to lateral pain (0.7 ± 0.8 degrees, *p* = 0.022). No other post-hoc comparisons were significant for hip or knee extension (multiple *p* > 0.096).

Main effects were also observed for peak hip adduction (F(4,80) = 5.086, *p* = 0.001) and knee flexion (F(2.7,54.8) = 3.707, *p* = 0.020). People walked with larger peak hip adduction during post compared to when pain was induced in the anterior (0.5 ± 0.6 degrees, *p* = 0.005) and lateral (0.5 ± 0.7 degrees, *p* = 0.023) locations. Larger knee flexion peak was observed when comparing post to baseline (1.4 ± 1.5 degrees, *p* = 0.003) and medial (1.1 ± 1.4 degrees, *p* = 0.023). Peak hip flexion approached statistical significance (F(4,80) = 2.468, *p* = 0.051). This main effect was driven by the fact that participants walked with larger hip flexion after the end of the painful stimulation protocol compared to baseline (0.7 ± 1.0 degrees, *p* = 0.030). No other post-hoc comparisons were significant (multiple *p* > 0.059).

The presence of pain did not alter ankle flexion kinematics, hip rotation kinematics, or peak hip adduction (multiple F(4,80) < 1.409, *p* > 0.239).

### Helical axes

Helical axes could not be estimated in one participant. In the remaining 20 participants, there was a general trend for an increase in variability of the helical axes during and after pain compared to baseline.

The mean distance of the helical axes on the sagittal plane was influenced by the presence of experimental pain during late stance (*χ*^2^(4) = 13.480, *p* = 0.009). This effect was mainly driven by a larger mean distance during anterior pain (0.7 [0.3, 1.4], *p* = 0.010) and post (0.8 [0.2, 1.2], *p* = 0.037) compared to baseline. A significant increase of mean distance (*χ*^2^(4) = 10.920, *p* = 0.027) was also observed during early swing when pain was induce medially compared to baseline (0.3 [0.1, 0.5], *p* = 0.037).

A significant effect of condition was also observed on the mean angle of the helical axes on the sagittal plane during early (F(4,76) = 5.12, *p* < 0.001) and late swing (F(4,76) = 2.705, *p* = 0.036). Post-hoc decomposition of these main effects revealed a larger mean angle in post compared to baseline both in early (1.1 ± 1.3, *p* = 0.009) and late (1.0 ± 1.4, *p* = 0.049) swing, and larger mean angle during medial pain compared to baseline in early (1.2 ± 1.4, *p* = 0.010) swing. No other post-hoc comparisons were significant (multiple *p* > 0.061) (Table 3).

**Table 3:** Helical axes on the sagittal plane

	Baseline	Medial	Anterior	Lateral	Post	*p*
Mean distance						
Stance 95–10%	3.3 [3.0, 4.1]	3.7 [3.2, 4.5]	3.5 [3.0, 4.3]	3.5 [3.0, 4.1]	3.5 [3.2, 4.1]	0.252
Stance 10–40%	3.8 [2.8, 4.3]	3.8 [3.1, 4.6]	4.5 [3.3, 5.2]	4.3 [3.3, 4.8]	4.3 [3.3, 5.4]	0.009; *AP* > *B*
Swing 40–75%	2.8 [2.5, 3.2]	2.9 [2.6, 3.6]	3.0 [2.7, 3.4]	3.2 [2.5, 3.4]	3.0 [2.8, 3.3]	0.027; *M* > *B*
Swing 75–95%	4.3 [3.9, 5.0]	5.3 [3.9, 6.2]	5.4 [4.0, 7.2]	5.4 [3.9, 6.6]	4.3 [3.4, 6.5]	0.565
Mean angle						
Stance 95–10%	13.6 ± 3.3	14.0 ± 3.4	14.8 ± 4.1	14.1 ± 3.0	14.2 ± 2.8	0.460
Stance 10–40%	12.6 [9.7, 14.9]	13.5 [10.5, 20.0]	15.7 [10.5, 20.7]	13.3 [11.6, 20.6]	14.5 [11.3, 17.1]	0.077
Swing 40–75%	11.4 ± 2.1	12.6 ± 2.0	12.8 ± 1.9	12.3 ± 2.3	12.5 ± 2.3	< 0.001; *MP* > *B*
Swing 75–95%	10.6 ± 2.1	11.1 ± 2.8	11.3 ± 2.6	11.2 ± 2.8	11.6 ± 2.7	0.036;* P* > *B*

*A* anterior, *B* baseline, *L* lateral, *M* medial, *P* post

## Discussion

In this study, we used surface electrical stimulation to investigate whether pain experimentally induced in the medial, lateral and anterior sides of the knee differentially affect gait kinematics and the variability of the knee helical axis. Anterior knee pain reduced knee and hip extension during the stance phase of gait cycle. Mean distance of the helical axis increased during anterior pain in the late stance phase of gait cycle, whereas medial pain resulted in larger mean distance and mean angle during early swing. Our findings suggest that acute, experimental knee pain alters both joint kinematics and arthrokinematics, and that motor adaptation depends on the spatial characteristics of the painful stimulus.

Pain drawings revealed that participants perceived pain in the medial, anterior, or lateral side of their knee, which demonstrates that electrical stimulation can be effectively used to compare the effect of pain induced in different locations. Compared to previous studies using multiple injections of hypertonic saline solution (Tsao et al. 2010; Tucker et al. 2014; Gallina et al. 2018; Oda et al. 2018), the main advantages of this model are that it is non-invasive, it can target tissues that are difficult to inject (e.g.: bone/periosteum), and that the pain intensity can be standardized to a target intensity. Limitations include lower spatial specificity and the impossibility to selectively target deep tissues. Participants reported a larger pain area for the anterior location compared to the medial and lateral locations, which reproduced well the spatial characteristics of the pain experience of individuals with knee osteoarthritis (Thompson et al. 2009). This was accomplished using small interelectrode distances on the medial and lateral side of the knee, and electrodes further apart on the patella. While previous research identified a fast habituation of perceived pain intensity during continuous stimulation for 1 min using square waves (Gallina et al. 2021), data from this study show only a non-significant decrease of pain ratings of approx. 0.5/10. This could be due to a number of methodological differences between the two studies, including task (gait vs rest), frequency of pain rating reporting (start and end vs every 10 s), electrode position (over the infrapatellar fat pad vs over the sides of the patella). We are currently unable to determine what the main determinant of these differences between studies is, therefore, this issue will need to be investigated in future studies with ad hoc protocols. Overall, our experimental approach was effective in inducing localized pain with spatial characteristics similar of that reported by individuals with clinical knee conditions.

An unexpected finding of our research is that, despite a constant painful electrical stimulus, participants reported constant pain throughout the gait cycle in less than 33% of the trials. The temporal patterns of pain intensity were highly variable across participants and pain locations, but when a participant repeated the same trial twice, similar temporal fluctuations of pain intensity were reported in almost 80% of the cases. This variability across participants and locations but consistency between trials may be explained by an interaction between the characteristics of the stimulation (e.g., electrode position) and the characteristics of the tissue stimulated (e.g., tissues sliding under the electrodes, stretch/compression of the stimulated tissue, and local variations in density of the nociceptors). Between-individuals variations in anatomy and tissue mechanics during walking with respect to the electrode placement may result in individual-specific, repeatable variations in pain intensity throughout the gait cycle despite constant electrical stimulation. Another possible contributor to this phenomenon is a sensitization to pressure stimuli. Lower pain pressure threshold has been observed in individuals with patellofemoral pain (Rathleff et al. 2013; Heijden et al. 2018), and when healthy individuals are exposed to experimental pain models such as hypertonic saline solution (Graven-Nielsen et al. 2003; Oda et al. 2018), nerve growth factor (Andersen et al. 2008; Bergin et al. 2015) and exercise-induced muscle damage (Hedayatpour et al. 2008). While it is currently unknown whether pressure pain threshold decreases during painful electrical stimulation, it is possible that an increased sensitivity to mechanical pressure may have contributed to the temporal differences in pain perception across the gait cycle.

Participants walked with reduced knee and hip extension when painful stimuli were applied to the anterior region of the knee. Our finding of reduced knee extension during the stance phase of gait is in line with some (Henriksen et al. 2010; Son et al. 2017) but not all (Seeley et al. 2013), studies where pain was induced by injecting or infusing hypertonic saline solution in the fat pad, and when joint effusion was induced experimentally (Torry et al. 2000). Reduced knee extension during gait is also common in individuals with tibiofemoral osteoarthritis (Henriksen et al. 2010; Mills et al. 2013). Our findings of unchanged knee extension when pain was induced medially or laterally concur with the lack of adaptation observed when pain was induced not in the anterior region of the knee, for instance in the vastus medialis (Henriksen et al. 2007) or in the hamstring (Henriksen et al. 2011b), suggesting that reduced knee extension during gait may be an adaptation specific for pain perceived in the anterior region of the knee. It should be noted that pain intensity was matched across pain locations (especially later during the trial), but anterior knee pain resulted in larger perceived pain area compared to pain induced in the medial or lateral location, therefore the larger pain area may also have contributed to the differences observed. It should be noted that the absolute changes in knee angle are small (1.5 degrees for knee extension, 0.7 degrees for hip extension); however, it is hypothesized that even subtle changes in kinematics may potentially result in uneven load distribution that may result in long-term consequences (Hodges and Tucker 2011). Our data also show that knee extension returned to baseline in the post-trial, implying that motor adaptation did not outlast pain duration as observed in other studies that induced pain around the knee (Tucker et al. 2012; Gallina et al. 2018). In the post-trial, individuals also walked with larger knee flexion. It is possible that these are natural adaptations to walking on a treadmill, which could not be detected during the trials when participants experienced pain. This hypothesis is, however, speculative at the moment, and further research is necessary to confirm or refute it.

Acute experimental pain resulted in an overall increase in variability of the helical axes in the sagittal plane, especially during early swing and late stance. While these values are small compared to the standard error of measurement reported in our reliability study (Adamo et al. 2022), those values referred to between-day reliability which included repositioning of the markers. Instead, in this study, we performed a within-session comparison without repositioning the markers, therefore, the standard error of measurements is likely to be smaller. Larger mean distance and mean angle have previously been interpreted as decreased joint stability and increased motor variability, due to altered motor control strategies or altered morphology of the joint (Grip et al. 2008; Temporiti et al. 2019, 2020; Alsultan et al. 2019; Cattrysse et al. 2020). Similar to what observed after fatigue (Adamo et al. 2020), our within-subject design supports the notion that altered motor control strategies are a main determinant of helical axes behavior. Our findings of increased variability are apparently in opposition with previous findings of reduced dispersion of neck helical axes in individuals with chronic neck pain (Alsultan et al. 2019; Cattrysse et al. 2020). However, the findings of the two studies can be integrated and interpreted according to current motor adaptation theories (Hodges and Tucker 2011), which predict an increase in motor variability in the short term (to find a motor solution that reduces pain), and a decrease of motor variability in the long term (when movement strategy that reduces pain has been identified). This hypothesized time course of motor adaptation to pain may explain the differences in helical axis behavior between the two studies, although other factors (experimental vs clinical pain, joint tested, etc.) may also play a role. Even in these arthrokinematics measures, the spatial characteristics of the painful stimulus played a role in determining the motor adaptation, with anterior pain increasing variability during late stance, and medial pain increasing variability mainly during early swing. Differently from joint kinematics, in some cases, the variability of the helical axes was still increased in the trials without pain, potentially supporting the notion that pain is a stimulus to adapt how someone moves, but pain resolution is not a stimulus sufficient to restore their original movement strategies.

This study has some limitations. First, we decided to test adaptation to pain induced in different locations in the same session to limit issues related to the between-day reliability of motion capture. Carry-over effects between the adaptation to pain induced in different locations may have resulted in increased variability in the data, which may have limited our ability to detect changes between pain locations. The randomization of pain locations and the lack of long-lasting kinematic adaptation in the post-trial support our approach, although the estimation of helical axis variability may have been more prone to this issue since some adaptations were observed even in the trial without painful stimulation. Second, surface electrical stimulation is known to be not specific. A risk in this study is that the adaptation is due to the activation of receptors other than nociceptors, such as skin, ligaments, and muscle. We have partially limited this by placing the stimulation electrodes away from the muscle and carefully assessing whether muscle twitches were visible during the stimulation. In addition, previous research demonstrated that reflexes from receptors in the medial collateral ligament cannot be elicited by surface stimulation (Kim et al. 1995). We were unable to reduce the contribution of skin non-nociceptive afferents. However, pain drawings demonstrate that participants perceived pain deeper than the skin, which suggests that the painful stimulation used in this study was able to target deeper nociceptors, and not exclusively skin receptors.

Overall, this study demonstrates that acute, experimental knee pain induces changes in gait kinematics and increases the helical axis dispersion, and that these adaptations depend on the spatial characteristics of the painful stimulus. The observed adaptations may reflect an attempt of the central nervous system to protect the painful tissue while searching for a less painful movement strategy.

## Data Availability

The datasets generated during and/or analyzed during the current study are available from the corresponding author on reasonable request.

## Abbreviation

ANOVA: Analysis of variance
